# Second WSES convention, WJES impact factor, and emergency surgery worldwide

**DOI:** 10.1186/1749-7922-8-15

**Published:** 2013-04-16

**Authors:** Fausto Catena, Massimo Sartelli, Luca Ansaloni, Frederick Moore, Ernest E Moore

**Affiliations:** 1 Department of Emergency Surgery, Parma University Hospital, Parma, Italy; 2 1st Department General Surgery, Pope John XXIIII Hospital, Bergamo, Italy; 3 Department of Surgery, Denver Health Medical Center and the University of Colorado Denver, Denver, CO, USA; 4 Department General Surgery, Macerata Hospital, Macerata, Italy; 5 Department of Emergency Surgery, University of Florida, Gainesville, FL, USA

## Background

In 2007, before founding the World Society of Emergency Surgery (WSES), we developed a questionnaire to investigate how emergency surgery was organized and implemented as a practice throughout the world. We discovered two fundamental models, which were sometimes present simultaneously in the same country [1].

One model was characterized by hospitals with designated emergency surgery departments and the other featured hospitals without an emergency surgery department in which surgical emergencies were subdivided among various general and specialized surgeons. Similarly, some hospitals had designated trauma teams while others had no such designated units.

However, despite the heterogeneous complexity of emergency surgery in a worldwide context, the work of surgeons around the globe appears remarkably similar regardless of the name attributed to the facility in which they practice, be it emergency surgery, acute care surgery, or another generic title.

Although it is difficult to succinctly define emergency surgery, which includes a broad spectrum of procedures, a universal definition could be poly-specialized surgery performed for traumatic and non-traumatic acute diseases. We have considered non traumatic emergency surgery as non CNS life-threatening diseases requiring urgent operative intervention (within 24 hr) with the exception of those requiring total cardiac bypass.

There is a significant difference between traumatic and non-traumatic acute diseases. The dispersion of trauma programs sponsored by the American College of Surgeons has resulted in the near-uniform management of trauma patients around the world. By contrast, the management of patients with non-traumatic acute diseases (intra-abdominal infections, bowel occlusion, etc.) remains poorly standardized and varies dramatically between treatment centers. Standards for the management of non-traumatic acute diseases are just as important as those of ATLS.

Practitioners of emergency surgery worldwide must develop standardized guidelines to streamline protocol and designate organizational models used to address acute diseases requiring urgent surgical intervention; this ambitious effort is the primary objective of the World Society of Emergency Surgery (WSES) and its publication affiliate the *World Journal of Emergency Surgery* (WJES)*.*

In recent years, the WSES has focused on non-traumatic acute diseases, proposing standardized protocol guidelines and prospective studies shared worldwide.

In 2011, WSES published the first set of universal guidelines for the management of intra-abdominal infections in the WJES [2]. This article was an executive summary of the final recommendations approved by the consensus conference held in Bologna, Italy, in July of 2010 during the first WSES convention. These guidelines were recently updated following a multidisciplinary collaboration of international contributors [3].

In 2011, the WSES also presented guidelines for the management of obstructive cancer of the left colon [4] as well as guidelines for the diagnosis and management of adhesive small bowel obstruction [5], both published in the WJES. These guidelines represent a summary of the final recommendations approved by the consensus conference of the first WSES convention held in Bologna in 2010.

In an effort to promote global sharing on the topic of management of intra-abdominal infections and to garner international support and input, the WSES also conducted two prospective observational studies.

The CIAO Study (“*C*omplicated *I*ntra-*A*bdominal infection *O*bservational” Study) was a multicenter investigation performed in 68 medical institutions throughout Europe over the course of a 6-month observational period (January-June 2012) [6].

Given the success of the CIAO Study, WSES designed a broader continuation of the study, a prospective observational investigation of the management of complicated intra-abdominal infections in a worldwide context

The CIAOW study (“*C*omplicated *I*ntra-*A*bdominal infection *O*bservational *W*orldwide” Study) is a multicenter observational study currently underway in 57 medical institutions around the world [7]. A comprehensive review of the CIAO Study and the preliminary results of the CIAOW study were published recently in the WJES [6,7].

The final project that we will discuss during the second WSES convention is the development of a triage system for cases of acute non-traumatic surgery.

The second WSES convention will be held in Bergamo, Italy, July 7–9, 2013 (http://www.mitcongressi.it/wses2013/). Experts of emergency surgery from around the world will discuss current research and findings throughout the three-day convention. The objective of the convention is to update the international surgical community on state-of-the-art advancements in emergency surgery and discuss how these advancements can be implemented in routine practice. Upon conclusion of the convention, all participants will undergo the Emergency Surgery Education Test to receive the WSES Emergency Surgery Diploma (ESD).

During the convention, we will also announce the official impact factor of the *World Journal of Emergency Surgery.*
